# Advances in Social Cognitive and Affective Neuroscience: Ten Highly Cited Articles Published in *Brain Sciences* in 2022–2023

**DOI:** 10.3390/brainsci14050460

**Published:** 2024-05-02

**Authors:** Yang Zhang

**Affiliations:** Department of Speech-Language-Hearing Sciences & Center for Neurobehavioral Development, University of Minnesota, Twin Cities, MN 55455, USA; zhanglab@umn.edu; Tel.: +1-612-624-7818

In the realm of Social Cognitive and Affective Neuroscience, researchers employ a variety of methods to address theoretical and practical questions that focus on the intricate interplay between social perception, cognition, and emotion across diverse populations and contexts [1,2,3,4]. They strive to examine the complexities of human behavior and unravel its neural underpinnings with behavioral and brain data from both clinical populations and healthy individuals. As advocates for open access to knowledge, we are excited to feature a selection of ten highly cited articles published over the last two years in *Brain Sciences*. These studies exemplify recent endeavors covering both breadth and depth within this field and offering valuable insights into the various aspects of human cognition, emotion, and social behavior.

**1.**  **Understanding Trauma and Attachment**

Fonagy, Campbell, and Luyten [5] provided a review of the current status of research on the relationship between attachment and trauma in developmental psychopathology. Their article examined major issues in attachment theory regarding trauma’s impact and intergenerational transmission and explored recent neurobiological findings on mentalization and trauma, offering implications for clinical practice. By integrating neurobiological perspectives, they illuminate the intricate dynamics between early attachment experiences and responses to trauma, shaping our understanding of developmental psychopathology.

**2.**  **Exploring Cognitive Interventions in Psychiatric Disorders**

Deste et al. [6] examined the efficacy of physical exercise and cognitive remediation, both independently and in combination, as interventions for cognitive impairment in schizophrenia. Their articles synthesized findings from nine meta-analyses and systematic reviews on physical exercise alone and discussed seven studies combining physical exercise with cognitive remediation. This critical review highlights the well-documented efficacy of physical exercise in improving cognitive performance in schizophrenia and suggests that integrated interventions offer superior benefits. However, it also emphasizes the need for further research to optimize intervention delivery and explore long-term effects that will help shape future psychiatric rehabilitation practices.

**3.**  **Unveiling Neurobiological Mechanisms in Clinical Conditions**

Pruneti and Guidotti [7] investigated neurovegetative activation in women with Functional Hypothalamic Amenorrhea (FHA). They compared 25 women with FHA to a control group to explore the physiological and psychological correlates of this condition. Through psychometric assessments and the Psychophysiological Stress Profile (PSP), the study revealed autonomic hyperactivation and psychological distress in FHA patients. These findings highlight the intricate interplay between stress, reproductive health, and mental well-being, emphasizing the need for comprehensive assessment and multidisciplinary interventions in FHA management.

**4.**  **Adapting to New Norms Amidst a Pandemic**

Biggio et al. [8] examined the impact of mask-wearing on interpersonal space during the COVID-19 pandemic. Through a reaching-comfort distance estimation task involving avatars with and without masks, their research revealed that interpersonal space was greater between individuals not wearing masks. These findings suggest a contextual adaptation of interpersonal space in response to pandemic-related norms that points to the psychological implications of protective measures on social behavior.

**5.**  **Investigating Sensory Processing and Dysfunction**

Yan et al. [9] examined the relationship between olfactory bulb (OB) shape and olfactory function. They used both MRI scans and olfactory testing and demonstrated that non-convex OB patterns were more common in patients with olfactory disorders. These findings demonstrate that OB shape is a potential biomarker for olfactory dysfunction, which offers diagnostic insights into olfactory disorders.

**6.**  **Enhancing Cognitive Function through Physical Activity**

Song et al. [10] conducted a review of studies that employed randomized controlled trials (RCTs) to assess the effects of cognitively engaging physical activity (PA) interventions on executive functions (EFs) in children aged 4 to 12. Their review analyzed data from 11 articles and identified improvements in overall EFs, updating, and shifting abilities following PA interventions. The convergent findings emphasize the effectiveness of such interventions in enhancing EFs, particularly updating and shifting, underscoring the importance of integrating physical activity into childhood education.

**7.**  **Promoting Cognitive Health in Aging**

Yamasaki’s [11] review article covered preventive strategies for cognitive decline and dementia through aerobic physical activity interventions and discussed the mechanisms underlying their effectiveness. Emphasizing aerobic physical activity and open-skill exercise, the review highlights their superior protective effects on cognitive function. The cumulative evidence in the literature supports the advocacy of physical activity interventions as a non-pharmaceutical strategy to preserving cognitive abilities in older adults.

**8.**  **Investigating Neural Correlates of Affective Processes**

Chang, Chan, and Chen’s [12] functional Magnetic Resonance Imaging (fMRI) study aimed to study the neural mechanisms of humor appreciation to validate the four-stage model of humor processing. Their study identified brain regions associated with each stage and proposed adding the expectation stage to the existing model. These findings enhance our understanding of the brain regions and mechanisms involved in humor processing in affective neuroscience.

**9.**  **Addressing Complex Comorbidities in Neurological Disorders**

Palermo et al. [13] investigated the interplay between Parkinson’s disease (PD), SARS-CoV-2 infection, and frailty. They proposed a multidimensional screening protocol for identifying individuals at risk. The results highlighted hypovitaminosis D as a potential link between frailty, COVID-19, and PD progression and showed the importance of multidisciplinary care in managing complex comorbidities. This study provides insights into potential mechanisms underlying disease progression and calls for proactive screening and intervention strategies.

**10.**  **Exploring Emotion and Creativity through Music**

Ramirez-Melendez and Reija [14] explored the emotional correlates of creative drumming using EEG recordings. By analyzing arousal and valence levels during different degrees of creative music playing, this study identified increased positive emotions during pattern-based and free improvisation. These findings suggest a link between positive emotion and creative expression in drumming, which points to the therapeutic potential of music-based interventions and music pedagogy.

Overall, these ten articles reflect some of the evolving and emerging trends in the field [15]. Neuroimaging researchers are increasingly adopting multimodal and multidisciplinary approaches to investigate the dynamic interactions in brain regions and networks implicated in social cognition and emotion regulation [16]. Clinical and translational research has played a pivotal role in informing and transforming the development and enhancement of psychotherapeutic interventions [17], which help modulate neural activity in the critical brain networks involved in the social cognitive and affective processes. There is a growing interest in understanding the development of social cognitive and affective processes across the lifespan [18], which includes studying how early social experiences shape neural circuits underlying empathy, social interaction, theory of mind and decision making. Computational models are gaining prominence for their capability to offer mechanistic insights into social information processing such as mentalizing (e.g., inferring other people’s thoughts and intentions) and social learning [19]. Some recent endeavors have also focused on exploring cultural and contextual factors in emotion cognition and social behavior [20]. These emerging trends carry significant implications for comprehending and addressing psychiatric disorders characterized by social cognitive and effective deficits such as autism spectrum disorder, schizophrenia, dementia, and mood disorders [21]. It is important to note that there are undoubtedly numerous other outstanding and impactful studies in the field that have contributed to our deeper understanding of the brain mechanisms underlying social cognition, emotion, and behavior.

In showcasing these highly cited articles in *Brain Sciences* that covered a wide range of topics, including attachment, mentalization, and trauma, cognitive function and psychiatric disorders with comorbidity conditions, neurobiology of sensory and music perception, social interaction and context-shaped behavior, cognitive development, and aging, we celebrate the collective endeavors of researchers and the multidisciplinary approaches pushing the boundaries of knowledge in Social Cognitive and Affective Neuroscience. These articles align well with the emerging directions and trends in the evolving landscape of this field. These findings and critical perspectives have opened up the door to innovative interventions and holistic approaches to mental health and well-being. As proponents of open science, we invite readers to explore these relatively new contributions and embark on a journey of discovery in understanding the intricacies of the human mind and behavior.
